# *Trifolium pratense* and *T. repens* (Leguminosae): Edible Flower Extracts as Functional Ingredients

**DOI:** 10.3390/foods4030338

**Published:** 2015-08-21

**Authors:** Rosa Tundis, Mariangela Marrelli, Filomena Conforti, Maria Concetta Tenuta, Marco Bonesi, Francesco Menichini, Monica Rosa Loizzo

**Affiliations:** Department of Pharmacy, Health and Nutritional Sciences, University of Calabria, Rende (CS) 87036, Italy; E-Mails: rosa.tundis@unical.it (R.T.); mariangela.marrelli@unical.it (M.M.); filomena.conforti@unical.it (F.C.); mary.tn2006@hotmail.it (M.C.T.); marco.bonesi@unical.it (M.B.); francesco.menichini@unical.it (F.M.)

**Keywords:** *Trifolium*, clover, flavonoids, antioxidant activity, α-amylase, α-glucosidase, lipase

## Abstract

*Trifolium pratense* (red clover) and *T. repens* (white clover) edible flowers were investigated for their chemical profile and health properties. The total phenols and flavonoids contents were evaluated. Quercetin, kaempferol, luteolin, rutin, and myricetin were used as markers and quantified by HPLC. The antioxidant effects were investigated by using different *in vitro* assays. Moreover, α-amylase, α-glucosidase and lipase inhibitory activities were evaluated. *T. repens* flowers extract showed a good radical scavenging activity in both DPPH and ABTS tests with IC_50_ values of 10.3 and 21.4 μg/mL, respectively. White clover extract demonstrated promising α-amylase and lipase inhibitory activities with IC_50_ values of 25.0 and 1.3 μg/mL, respectively. The obtained results support the use of *Trifolium* flowers as healthy food ingredients.

## 1. Introduction

*Trifolium* is one of the most important genera of the Leguminosae family [1]. *Trifolium* species are generally known as clover. Its flowers have a sweet and mild licorice flavor and are traditionally used garnish or ingredient in salads, soups, entrees, desserts, and drinks worldwide [2]. They are used not only to improve appearance of meals but also for their nutritive value [3].

Polyphenols are phytochemicals generally involved in defense against ultraviolet radiation or aggression by pathogens. Epidemiological studies suggest that long term consumption of diets rich in plant polyphenols offer protection against chronic disease such as diabetes and obesity. In food, polyphenols may contribute to the bitterness, astringency, color, flavor, odor and oxidative stability [4].

Diabetes and obesity are the biggest public health challenge of the 21st century. Type 2 diabetes or non-insulin-dependent or adult-onset results from the body’s ineffective use of insulin or as consequence of low amounts of insulin production from pancreatic β-cells or as peripheral insulin resistance [5]. 

About 80% to 90% of patient with type II diabetes are also diagnosed as obese. This fact provides interesting evidence of the relationship between obesity and diabetes. Being overweight places extra stress on your body in a variety of ways, including your body’s ability to maintain proper blood glucose levels [6,7]. In diabetic patients, free radicals are formed by glucose oxidation, glycation of proteins, and their oxidative degradation. Abnormally high levels of free radicals and the simultaneous decline of antioxidant defense mechanisms can lead to damage of cells, increased lipid peroxidation, and development of insulin resistance and the development of diabetic complications [8]. For this reason, molecules with antioxidant potential may be useful for the adequate maintenance of oxidative levels in blood. One therapeutic approach for management of diabetes type 2 is to decrease post-prandial hyperglycaemia through the inhibition of the carbohydrate-hydrolysing enzymes, α-amylase and α-glucosidase [9]. The α-amylase enzyme is produced by the pancreas and it is also found in saliva while the α-glucosidase acts in the mucosal brush border of the small intestine [10]. Drugs able to inhibit both enzyme such as acarbose, miglitol and voglibose are extensively prescribed; however, they are characterized by several side effects (e.g., bloating, diarrhea, gas, and stomach pain) [11]. Patients with diabetes type 2, especially if they are also obese, are affected also by plasma lipid and lipoprotein abnormalities, which include reduced HDL cholesterol, a predominance of small dense LDL particles, and elevated triglyceride levels. The lipid changes associated with diabetes mellitus are attributed to increased free fatty acid flux secondary to insulin resistance [12]. Diabetes seems to influence the cholesterol absorption efficiency and synthesis with the respective non diabetic state. For this reason, diabetic patients are often affected by high levels of cholesterol in the blood [13]. Pancreatic lipase is a key enzyme involved in dietary fat digestion. Interference with fat hydrolysis results in the reduced utilization of ingested lipids, therefore inhibition of lipases decreases fat absorption that is useful for obese patients [14]. Orlistat, a hydrogenated derivative of lipstatin, is the only pancreatic lipase inhibitor currently approved for a long-term treatment of obesity. Several natural products are able to inhibit key carbohydrate-hydrolysing enzymes [15,16,17] and also pancreatic lipase [18,19]. In this work, edible flowers from two different *Trifolium* ssp (*T. repens* and *T. pratense*) were *in vitro* investigated for their chemical composition, antioxidant activity, hypoglycaemic potential by inhibition of α-amylase and α-glucosidase, and pancreatic lipase inhibitory properties.

## 2. Experimental Section

### 2.1. Chemicals and Reagents

α-Amylase from porcine pancreas (EC 3.2.1.1), α-glucosidase from *Saccharomyces cerevisiae* (EC 3.2.1.20), anhydrous sodium sulfate, maltose, sodium acetate, potassium hydrogen carbonate, sodium potassium tartrate, 3,5-dinitrosalicylic acid, *o*-Dianisidine Color Reagent (DIAN), peroxidase/glucose oxidase (PGO) solution, potato starch, sodium phosphate, sodium chloride, acetic acid, perchloric acid, basic bismuth nitrate, potassium iodide, phosphoric acid, sodium hydroxyde, HEPES (4-(2-hydroxyethyl)-1-piperazineethanesulfonic acid), sodium phosphate monobasic, chlorogenic acid, quercetin, kaempferol, luteolin, rutin, myricetin, sodium phosphate buffer, 2,2-diphenyl-1-picrylhydrazyl (DPPH), Folin-Ciocalteau reagent, Tween 20, tripyridyltriazine (TPTZ), sodium potassium tartrate tetrahydrate, 2,20-azino-bis(3-ethylbenzothiazoline-6-sulphonic acid (ABTS) solution, 6-hydroxy-2,5,7,8-tetramethylchroman-2-carboxylic acid (Trolox), potassium persulphate, β-carotene, Tween 20, linoleic acid, FeCl_3_, FeSO_4_, butylatedhydroxytoluene (BHT), ascorbic acid, propyl gallate, cyanidin-3-glucoside, Lipase Type II from porcine pancreas (EC 3.1.1.3), 4-nitrophenyl octanoate, and orlistatwere purchased from Sigma-Aldrich S.p.a. (Milan, Italy). Acarbose from *Actinoplanes* sp. was obtained from Serva (Heidelberg, Germany). Solvents of analytical grade were obtained from VWR International s.r.l. (Milan, Italy).

### 2.2. Extraction Procedure

*Trifolium repens* and *T. pratense* flowers were obtained from a market in Cosenza (Calabria, Italy) during spring 2013. Samples were cleaned by using distilled water; the petals were separated and kept at room temperature to drain. Then, flowers were extracted at room temperature by ethanol (48 h × 3 times) and evaporated to obtain the total extract (Table 1).

**Table 1:** Quantitative analysis by HPLC of selected flavonoids of *Trifolium repens* and *T. pratense* flowers extracts.

Edible Flowers	Rutin	Quercetin	Luteolin	Kaempferol	Myricetin
*Trifolium pratense*	-	-	16.7 ± 0.8	0.8 ± 0.02	0.5 ± 0.1
*T. repens*	45.8 ± 1.1	10.3 ± 0.2	-	0.5 ± 0.03	1.4 ± 0.02

Data represent means ± SD (standard deviation) (*n*= 3). Data are expressed as mg/g.

### 2.3. Determination of Total Phenols and Flavonoids Content

The total phenols content was determined by the Folin-Ciocalteau method [20]. The sample was mixed with 0.2 mL Folin-Ciocalteau reagent, 2 mL of water and 1 mL of 15% Na_2_CO_3_. After 2 h of incubation at 25 °C the absorbance was measured at 765 nm by using a UV-Vis Jenway 6003 spectrophotometer. The total phenols content was expressed as mg of chlorogenic acid equivalents per g of dry extract (Table 1).

The flavonoids content was determined as previously described by Yoo, *et al*. [21]. The levels of total flavonoids content were expressed as mg of quercetin equivalents *per* g of dry extract.

### 2.4. HPLC Analysis of Selected Flavonoids as Marker

Apigenin, kaempferol, luteolin, quercetin, and rutin were selected as markers and analysed by HPLC as previously described [14]. The HPLC system HP 1100 equipped with a pump, UV-vis detector (280 nm), column oven, injector and a C18 RP column (Phenomenex Luna 5 μm C18, 250 × 4.60 mm) was used. The mobile phase was H_2_O/formic acid (0.1%) (A) and methanol (B) with a flow rate of 1 mL/min (2 min 100% A; 8 min 80% A; 55 min 100% B; 65 min 100% A).

### 2.5. Antioxidant Activity

#### 2.5.1. Radical Scavenging (DPPH and ABTS) Activity Assays

The radical scavenging was investigated by ABTS and DPPH tests. ABTS radical cation (ABTS^·+^) solution was mixed with potassium persulphate and left in the dark for12 h before use. The ABTS^·+^ solution was diluted with ethanol to an absorbance of 0.70 ± 0.05 at 734 nm. After addition of extract or Trolox to the ABTS^·+^ solution, the absorbance was measured [22]. A DPPH ethanol solution (1.0 × 10^−4^ M) was mixed with sample at different concentration allow incubate in the dark for 30 min. The absorbance was measured at 517 nm. Ascorbic acid was used as positive control [23].

#### 2.5.2. β-carotene Bleaching Test

The β-carotene bleaching test was done following the procedure described by Loizzo *et al*. [24]. Briefly, a β-carotene solution was added to linoleic acid and 100% Tween 20. The emulsion was mixed with samples (200 μL) at different concentrations. The tubes were placed at 45 °C in a water bath for 60 min. The absorbance was measured at 470 nm at *t* = 0, 30 and 60 min. Propyl gallate was used as positive control.

#### 2.5.3. Ferric Reducing Activity Power (FRAP) Assay

The FRAP test is based on the redox reaction that involves TPTZ (2,4,6-tripyridyl-*s*-triazine)-Fe^3+^ complex [25]. FRAP reagent was prepared by mixing 10 mM TPTZ solution with 40 mM HCl, 20 mM FeCl_3_ and 0.3 M acetate buffer. The absorption was measured at 595 nm. The FRAP value represents the *ratio* between the slope of the linear plot for reducing Fe^3+^-TPTZ reagent by extracts compared to the slope of the plot for FeSO_4_. BHT was used as positive control.

### 2.6. *α*-Amylase and *α*-glucosidase Inhibitory Activity

The α-amylase and α-glucosidase inhibition assays were previously described by Loizzo *et al*. [26]. A mixture of α*-*amylase solution and samples at different concentrations was prepared and added to starch solution (25 °C for 5 min). The generation of maltose was quantified at 540 nm by using 3,5-dinitrosalicylic acid. In α-glucosidase assay samples at different concentrations were mixed with a maltose solution (*t* = 5 min at 37 °C). The α-glucosidase solution (10 units/mg) was added and left to incubate at 37 °C for 30 min. Then, the perchloric acid solution was added to stop the reaction. The generation of glucose was quantified by the reduction of DIAN at 500 nm.

### 2.7. Pancreatic Lipase Inhibitory Activity

The pancreatic lipase inhibition assay was performed as previously reported [27]. Porcine pancreatic lipase was mixed with water in order to obtain a solution with concentration of 1 mg/mL. Then a 5 mM solution of nitrophenyl caprylate in DMSO was prepared. The reaction mixture was prepared by adding 4 mL of Tris-HCl buffer (pH of 8.5), 100 mL of 5 mM nitrophenyl caprylate, 100 mL of sample and 100 mL of enzyme solution. Before adding the substrate the mixture was incubated at 37 °C for 25 min. The absorbance was measured at 412 nm. Orlistat was used as positive control.

### 2.8. Statistical Analysis

The concentration giving 50% inhibition (IC_50_) was calculated by nonlinear regression with the use of Prism GraphPad Prism version 4.0 for Windows (GraphPad Software, San Diego, CA, USA). The concentration-response curve was obtained by plotting the percentage inhibition *versus* concentration. Differences within and between groups were evaluated by one-way analysis of variance test (ANOVA) followed by a multicomparison Dunnett’s test compared with the positive controls.

## 3. Results and Discussion

### 3.1. Total Phenols and Flavonoids Content and HPLC Profile

*T. repens* exhibited the highest phenols and flavonoids content with 79.2 mg chlorogenic acid/g extract and 19.4 mg of quercetin equivalents/g of extract, respectively.

HPLC analyses of flavonoids (Table 1) revealed that *T. repens* contain high level of rutin (45.8 mg/g dry extract) followed by quercetin (10.3 mg/g dry extract). Kaempferol and myricetin are less expressed with values of 0.5 and 1.4 mg/g dry extract, respectively. *T. pratense* extract contain luteolin as main compound (16.7 mg/g dry extract) followed by kaempferol (0.8 mg/g dry extract) and myricetin (0.5 mg/g dry extract).

Recently, Xiong *et al.* [28] evaluated the flavonoids profile of ten common edible flowers from China and according with our results identified rutin and quercetin as the main abundant compounds.

### 3.2. Antioxidant Activity

The antioxidant potential of red and white clove extract was investigated by using different *in vitro* methods stable free radical scavengers: ABTS and DPPH; lipid oxidation β-carotene bleaching test and FRAP test. The use of a multiple approach is strongly recommended with food matrix considering that plant foods contain many different classes and types of antioxidants and that extraction procedure strongly influences the phytochemical composition of the extracts and, therefore, influence the antioxidant effects [29].

The radical scavenging ability investigated trough ABTS and DPPH methods evidenced that *T. repens* exhibited the highest potency with IC_50_ values of 21.4 and 10.3 μg/mL, respectively (Table 2). Significant DPPH scavenging activity was also observed with *T. pratense* (IC_50_ value of 34.0 μg/mL).

**Table 2:** Antioxidant activity of white and red clover flowers extracts.

	ABTS Test IC_50_ (μg/mL)	DPPH Test IC_50_ (μg/mL)	β-carotene Bleaching Test IC_50_ (μg/mL)		FRAP Test μM Fe(II)/g
Edible flowers			30 min	60 min	
*T. pratense*	149.8 ± 4.2 ***	34.0 ± 1.6 ***	7.9 ± 2.9 ***	11.0 ± 1.1 ***	NA
*T. repens*	21.4 ± 2.5 ***	10.3 ± 1.2	16.1 ± 2.4 ***	18.4 ± 2.8 ***	44.2 ± 4.5 ***
Positive controls					
Ascorbic acid	1.7 ± 0.3	5.0 ± 0.8			
Propyl gallate			1.0 ± 0.04	1.0 ± 0.05	
BHT					63.2 ± 4.3

Data are expressed as mean ± S.D. (*n* = 3). One-way ANOVA *** *p* < 0.0001 followed by a multicomparison Dunnett’s test: *** *p* < 0.01 compared with positive control. NA: not active.

Previously, Vlaisavljevic *et al.* [30] demonstrated that *T. pratense* essential oil showed a DPPH radical scavenging ability with an IC_50_ value 27.61 μg/mL.

Since lipid peroxidation in the body is primarily the oxidative damage of cell membranes, as well as all other systems that contain lipids, in determining the overall antioxidant activity of different compounds, it is necessary to examine their effect on the lipid peroxidation [31]. The effect of natural products on lipid peroxidation can be investigated by using different approaches and a number of different substrates (liposomes, linoleic acid, microsomes, various fatty oils, liver homogenate).

In the β-carotene bleaching test the oxidation of linoleic acid generates peroxyl free radicals due to the abstraction of a hydrogen atom from diallylic methylene groups of linoleic acid. The presence of phytochemical with antioxidant potential can hinder the extent of β-carotene bleaching by neutralizing the linoleate free radical and other free radicals formed in this model [32].

*T. pratense* showed a promising inhibition of lipid peroxidation with IC_50_ values of 7.9 and 11.0 μg/mL at 30 and 60 min of incubation, respectively. A lower protection on the β-carotene oxidation was observed when *T. repens* was applied in the model (IC_50_ values of 16.1 and 18.4 μg/mL at 30 and 60 min of incubation, respectively).

The reducing potential of phytonutrients contained in both red and white clover was investigated by using FRAP test. Generally, the reducing abilities related with the presence of molecules able to break the free radical chain through donating a hydrogen atom [33]. The ferric reducing ability powers of edible flower extracts expressed as FRAP values μM Fe(II)/g are shown in Table 2. *T. repens* flower extract exhibited a promising ferric reducing power with FRAP value of 44.2 μM Fe(II)/g.

### 3.3. Inhibition of Key Enzyme Involved in Diabetes and Obesity

The lowering of post-prandial hyperglycaemia through the inhibition of key-enzymes linked to type 2 diabetes mellitus is a critical and often used therapeutic approach. In this work, the ability of red and white clover flower extracts to inhibit α-amylase and α-glucosidase enzymes was investigated and results are reported in Table 3. All extracts exhibited hypoglycaemic activity in a concentration-dependent manner. *T. repens* extract showed the highest activity against α-amylase with an IC_50_ value of 25.0 μg/mL. This value is 2-fold lower than the commercial drug acarbose used as positive control (IC_50_ value of 50.0 μg/mL). The same extract showed also a promising activity against α-glucosidase with an IC_50_ value of 69.5 μg/mL. A comparable activity against α-glucosidase was observed with *T. pratense* extract.

**Table 3:** Inhibitory activity (IC_50_ (μg/mL)) of *T. pratense* and *T. repens* extract against key enzymes linked to diabetes and obesity.

Edible Flowers	α-Amylase	α-Glucosidase	Lipase
*T. pratense*	78.7 ± 2.1 ***	70.8 ± 3.9 ***	2.4 ± 0.1 ***
*T. repens*	25.0 ± 2.9 ***	69.5 ± 3.1 ***	1.3 ± 0.2 ***
Positive control			
Acarbose	50.0 ± 2.8	35.5 ± 1.2	
Orlistat			0.018 ± 0.005

Data are expressed as mean ± S.D. (*n* = 3). One-way ANOVA *** *p* < 0.0001 followed by a multicomparison Dunnett’s test: ***p < 0.01 compared with positive control.

The hypoglycaemic potential of *Trifolium* flower extracts could be ascribed on their flavonoids content since it is well documented that these phytochemical may act as α-amylase inhibitors [34].

Yuan *et al*. [35] demonstrated that quercetin and luteolin, two flavonoids identified in our samples, may act as non-competitive inhibitors of α-amyalse and that luteolin is more potent than quercetin and rutin. Both compounds are also able to inhibit α-glucosidase with IC_50_ values of 7 and 21 μM for quercetin and luteolin, respectively [36]. Rutin, the glycosylated conjugate of quercetin, had a stronger inhibition of α-amylase (IC_50_ value of 0.043 μM) and α-glucosidase (IC_50_ value of 0.037 μM) than quercetin.

Body mass index has a strong relationship to diabetes type 2 and insulin resistance. For this reason a multiple approach with compounds able to induce hypoglycaemia and reduce the fat absorption is desirable. In this work, we have screened the two *Trifolium* species for their ability to inhibit lipase. Both showed a promising lipase activity with IC_50_ values of 1.3 and 2.4 μg/mL for *T. repens* and *T. pratense*, respectively (Table 3). Previously, ‎AlRawi *et al*. [37] investigated the effect of different extracts of *Trifolium alexandrinum* flowers in a diabetes *in vivo* model. Extract improve the status of liver in streptozotocin-induced diabetic rats with a potency that follow this order water >*n*-hexane > ethanol. More recently, Aly *et al*. [38] demonstrated that the administration of a semi-modified diet containing 10% of *T. alexandrinum* ad-libitum for 6 weeks streptozotocin-induced diabetic rats induced a reduction of triglyceride, total cholesterol, LDL-cholesterol, and VLDL-cholesterol. These effects may be due to the regeneration of β-cells of the pancreas and potentiating of insulin secretion from surviving cells. The increase in insulin availability may lead to inhibition of lipid per oxidation and control of lipolytichormones. Chedraui *et al*. [39] investigated the *T. pratense* derived isoflavone supplementation for 90 days on the lipid profile of postmenopausal women with increased body mass index. The 88.3% of participants that completed the trial evidenced a significant decrease in total cholesterol, low-density lipoprotein cholesterol and lipoprotein A levels.

## 4. Conclusions

Obesity is the leading cause of major diseases including type 2 diabetes. At present, the potential use of natural products for the treatment of obesity is still largely unexplored and it might be an excellent alternative strategy for the development of safe and effective anti-obesity drugs. In this study, we have investigated the crude extracts of *T. repens* and *T. pratense* flowers. Our *in vitro* experiments showed that both species, rich source of flavonoids, are able to inhibit key enzymes involved in carbohydrate digestion such as α-amylase and α-glucosidase. An anti-lipase activity was also observed.

Plant foods generally contain flavonoids as glycosides. However, these phytochemicals undergo *in vivo* to a rapid metabolism by epithelial β-glucosidases [40]. The individual variability in the activity of these enzymes may be a factor determining variation in flavonoids bioavailability and consequently bioactivity. For the above-mentioned reason, further *in vivo* studies, which evaluate digestion, absorption and metabolism of the putative bioactive compounds, are necessary to establish the healthy properties of these two *Trifolium* species.
